# Effect of rabeprazole on the transport and distribution of levofloxacin in rat stomachs

**DOI:** 10.3892/etm.2014.2031

**Published:** 2014-10-17

**Authors:** JUNJUN BAO, YONGMEI HU, QIAO MEI, HAILUN ZHEN, JIANMING XU

**Affiliations:** Department of Gastroenterology, First Affiliated Hospital of Anhui Medical University, The Key Laboratory of Digestive Disease in Anhui Province, Hefei, Anhui 230022, P.R. China

**Keywords:** levofloxacin, *Helicobacter pylori*, transport, distribution, clearance, transport fraction

## Abstract

The aim of this study was to determine the transport and distribution characteristics of levofloxacin in the rat stomach and investigate the effects of combination treatment with rabeprazole. A total of 160 Wistar rats were randomly divided into four treatment groups: 50 mg/kg levofloxacin, 100 mg/kg levofloxacin, 50 mg/kg levofloxacin + rabeprazole and 100 mg/kg levofloxacin + rabeprazole. For two hours after intravenous administration, serum, gastric juice and stomach mucosa samples were collected at 15-min intervals, and the levofloxacin concentrations in all the samples were measured to determine the transport and distribution characteristics of levofloxacin in the rat stomach. In the 50 mg/kg levofloxacin and the 100 mg/kg levofloxacin groups, the drug concentration in the gastric juice gradually exceeded the serum concentration within 45–60 min of administration (P<0.05) and the drug concentrations in the gastric body and antrum were higher than those in the serum and the forestomach (P<0.05). At 15–30 min after administration, the drug concentrations in the gastric juice in the 50 mg/kg levofloxacin + rabeprazole and the 100 mg/kg levofloxacin + rabeprazole groups gradually exceeded the serum concentration (P<0.05). However, the levofloxacin concentration in the gastric body and in the antrum did not significantly differ between the two groups (P>0.05). The levofloxacin concentrations in each stomach region in the groups also treated with rabeprazole were higher than those treated with levofloxacin alone, but the differences were not significant. The levofloxacin transport fractions in the stomach in the 50 mg/kg levofloxacin, 100 mg/kg levofloxacin, 50 mg/kg levofloxacin + rabeprazole and 100 mg/kg levofloxacin + rabeprazole groups were 2.36, 2.52, 2.42 and 2.55, respectively, and no significant difference was identified. Levofloxacin may be actively transported in the rat stomach. The levofloxacin concentration in the gastric antrum exceeded that in the forestomach, and the local concentration increased with increasing dosage. Combining a proton pump inhibitor with levofloxacin has little effect on the concentration and distribution of levofloxacin in the stomach within 2 h.

## Introduction

*Helicobacter pylori* is the most important causative agent of various digestive diseases. The eradication of *H. pylori* using antimicrobial drugs has clinical implications (1–3). Based on pharmacological analysis, successful treatment with antimicrobial drugs depends on the drug concentration at the site of infection. The specificity of *H. pylori* colonises the gastric mucus and the surface of the mucosa (4), where the drug concentration should be sufficient to eliminate *H. pylori*. Intragastric delivery of antibiotics is a special characteristic of pharmacokinetics. Following drug intragastric distribution and intestinal absorption, antibiotics are delivered by blood to transmembrane transport of gastric tissue. Delivery of antimicrobial drugs to the stomach through the bloodstream decides the increase in the local concentration of the drug in the stomach, which is the key to the successful eradication of *H. pylori* (5,6). Proton pump inhibitor (PPI) antibiotic combined therapy (combination of clarithromycin, amoxicillin and metronidazole), the classical triple therapy, is the traditional option to eradicate *H. pylori*. Consequently, earlier studies on the intragastric delivery and distribution of antibiotics are mainly characterised by the diffusion and delivery of clarithromycin, amoxicillin and metronidazole (7,8,9). Previous experiments have indicated that the intragastric delivery of clarithromycin is becoming increasingly significant (7,9,10). Numerous clinical experiments have indicated that clarithromycin is the most effective drug for eradicating *H. pylori* (7), and that PPIs increase clarithromycin concentrations in the gastric tissue and mucus (11). These experimental results further explain the importance of antibiotic delivery via the bloodstream to the stomach and provide a theoretical basis for the classical option of antibiotics.

In recent years, the drug resistance of *H. pylori* has become more prominent, and the failure rate of the classical triple therapy is increasing (12,13). Thus, finding other vital remedial treatment options is necessary. Treatment with levofloxacin is among the primarily recommended drugs. Although levofloxacin-resistant strains have been found, the resistance rate is lower than that for clarithromycin and metronidazole. Triple therapy with levofloxacin remains a recommended alternative treatment option in the Guidelines of the American College of Gastroenterology (14), the 2012 Maastricht IV/Florence Consensus Report (12) and the Chinese Society of Gastroenterology National Consensus Report (13); thus, this strategy has been widely adopted. However, few studies have investigated the intragastric delivery and distribution of levofloxacin, and data on its pharmacokinetics via the stomach are insufficient. Thus, the intragastric delivery and distribution of levofloxacin require further study. Based on animal models, the present study initially investigated the intragastric delivery and distribution of levofloxacin in the rat stomach to determine the effects of its combination with rabeprazole on the delivery and distribution of levofloxacin, and to provide an advantageous experimental basis for the clinical application of levofloxacin for eradicating *H. pylori*.

## Materials and methods

### Animals

A total of 160 male Wistar rats weighing 250–300 g were obtained from Nanjing Medical University (Nanjing, China) and randomly divided into four dose groups: Levofloxacin (Double-Crane Pharmaceutical Co., Ltd., Beijing, China) at 50 mg/kg, levofloxacin at 100 mg/kg, levofloxacin at 50 mg/kg + rabeprazole (Jiangsu Aosaikang Pharmaceutical Co., Ltd., Nanjing, China) and levofloxacin at 100 mg/kg + rabeprazole. A dose of 50 mg/kg of levofloxacin in rats is equivalent to a human dose of 500 mg/day and 100 mg/kg in rats is equivalent to a human dose of 1,000 mg/day. This study was carried out in strict accordance with the recommendations in the Guide for the Care and Use of Laboratory Animals of the National Institutes of Health. The animal use protocol was reviewed and approved by the Institutional Animal Care and Use Committee of the First Affiliated Hospital of Anhui Medical University (Hefei, China).

The experimental period was 2 h with time-points every 15 min, for a total of eight time-points. At three days prior to the experiment, the rats were given a low-residue diet. The rats were then fasted but allowed water prior to the surgery. The animals were anaesthetised immediately prior to the procedure by injecting 3 ml/kg 10% chloral hydrate into the abdomen. The rats were fixed upon anaesthesia, and an indwelling choledochus tube was inserted to collect bile after laparotomy (to prevent bile reflux) (10). A monopolar electrotome (Changzhou Yanling Electronic Equipment Co., Ltd., Changzhou, China) was used to cut poorly vascularised sections of the duodenum 2 cm from the pylorus, and a rubber tube was inserted into the stomach via an incision between the duodenum and pylorus. Physiological saline was used to wash thoroughly the contents of the stomach until no residue was left in the absorbed liquid. The rats in each group were infused with levofloxacin/rabeprazole from the peripheral veins at their respective doses and routes of administration. At 15, 30, 45, 60, 75, 90, 105 and 120 min, blood was collected through the abdominal aorta of a rat in each group and the rat was sacrificed by cervical vertebra dislocation. The gullet and duodenum were immediately ligated and the gastric tissue was rapidly excised subsequent to cleaning the external section of the gastric tissue with physiological saline. The gastric juice was collected. The gastric tissues were sectioned along the longitudinal axis from the lesser curvature of the stomach, and the gastric mucosa was stripped carefully from the forestomach, gastric body and antrum. Subsequent to cleaning and weighing, the stripped stomach mucosa was homogenised in 1:9 volume of physiological saline. Once all the gastric juice samples and the diluent of the gastric mucosa homogenate had been assessed using occult blood test strips, the weakly positive or equivocally positive samples were collected.

The blood samples collected at each time-point were centrifuged for 10 min at 5,120 × g, and the supernatant was collected and stored at −80°C for later use. The bile capacity of the stomach samples collected at each time-point was accurately measured, and the samples were centrifuged for 10 min at 5,120 × g. The supernatant was collected and stored at −80°C for later use.

### High-performance liquid chromatography (HPLC)

HPLC analyses were performed using a Shimadzu LC-20AT pump equipped with a SPD-20AV variable-wavelength UV detector, an SIL-20A autosampler, a CTO-20AC thermostat and an LC solution system (Shimazu Co., Kyoto, Japan) (15). A Kromosil C18 column (250 ×4.6 mm, 5 μm; Shimadzu Co.) was used as the chromatographic column with a C18 Guard Precolumn (Phenomenex, Torrance, CA, USA), and the temperature was maintained at 50°C. The mobile phase consisted of 50 mmol/l acetonitrile, 1 mol/l citric acid and ammonium acetate (19:80:1, v/v). The detection wavelength was 295 nm, with 0.01 AUFS for all samples. The flow rate of the mobile phase was 1.0 ml/min, and the injection volume was 20 μl. The total running time was 6 min (16).

The linearity of the levofloxacin (standard purchased from the National Institutes for Food and Drug Control, Beijing, China) was investigated between 1 and 100 μg/ml in serum, gastric juice and gastric mucosa. Only 100 μl sample was required for detection. Subsequently, 100 μl 100 g/l trichloroacetic acid or methanol was individually added into the serum and the other samples to precipitate the proteins. The mixture was vortexed for 2 min and centrifuged for 10 min at 9,180 × g. The filtrates were obtained and 20 μl was detected for the drug concentration. Selectivity, linearity, accuracy, precision, stability and sensitivity were evaluated for method validation.

### Pharmacokinetics

The drug concentration-time curve was obtained based on the serum concentration assessed at each time-point from each experimental group of rats. The serum concentration, area under the curve (AUC), blood clearance, peak time and peak concentration in each group were calculated and compared.

The drug concentration-time curve was obtained according to the drug concentration of gastric juice in different dose groups at each time-point. The gastric mucosa of the different sections was also obtained. Separately, the drug concentrations of the gastric juice and mucosa in different dose groups were compared at each time-point. The relevant stomach clearance rate and transport fraction of the drug in the stomach were calculated using the following formulae (9,17):

$$\begin{array}{l} \text{Gastric clearance}=\frac{\text{Amount of drug transferred into gastric juice }(0.2h)}{\text{AUC }(0-2h)}\\ \text{Gastric Transfer Fraction}=\frac{\text{Gastric Clearance}}{\text{blood clearance}} \end{array}$$

### Statistical analysis

The statistical data were analysed by SPSS 16.0 statistical software (SPSS, Inc., Chicago, IL, USA). Difference comparisons of measurement data among the groups were determined using single-factor variance analysis. Comparisons between two groups were performed with a least significant difference Student’s t-test (α=0.05). P<0.05 was considered to indicate a statistically significant difference.

## Results

### HPLC method

The linearity of the levofloxacin was investigated between 1 and 100 μg/ml in the serum, gastric juice and gastric mucosa. The results are expressed as the correlation coefficient of determination (r^2^). The r^2^ of the samples was 0.999, which shows good linearity within the concentration range tested. The accuracy, precision and stability parameters are shown in Tables I–III.

### Comparison of distribution concentration for levofloxacin

**Figure 1:**
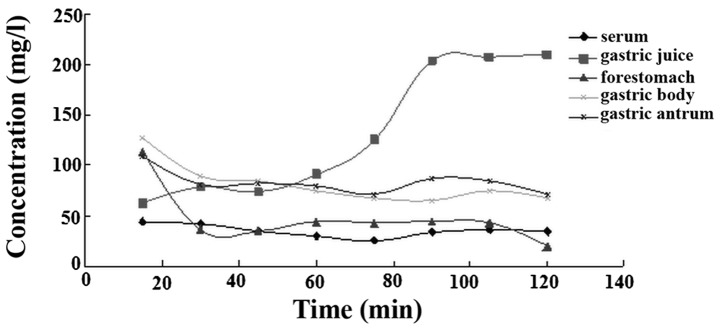
Mean concentration-time profiles of levofloxacin in the serum, gastric juice and all regions of the stomach at a 100 mg/kg dose in rats.

**Figure 2:**
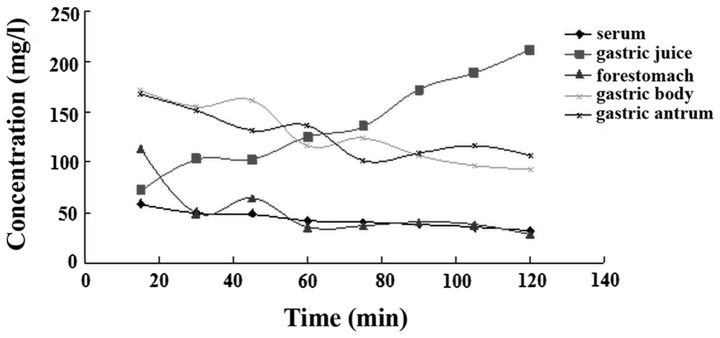
Mean concentration-time profiles of levofloxacin in the serum, gastric juice and all regions of the stomach at a 50 mg/kg dose in rats.

As shown in Figs. 1 and 2, the gastric juice levofloxacin concentration gradually increased starting from 60 min (100 mg/kg group) and 45 min (50 mg/kg group), at levels higher than the serum concentration. Starting from 15 min, the levofloxacin concentration in the gastric body and antrum was higher than the serum and forestomach levofloxacin concentration. Comparison of the levofloxacin concentration between the gastric body and gastric antrum revealed no difference (with the exception of the 15-min time-point), and the forestomach levofloxacin concentration exhibited no difference from the serum concentration. The stomach clearance rate of levofloxacin (100 mg/kg) was 1.18 l/h/kg, and the transport fraction was 2.52. The stomach clearance rate of levofloxacin (50 mg/kg) was 1.13 l/h/kg, and the transport fraction was 2.36.

**Figure 3:**
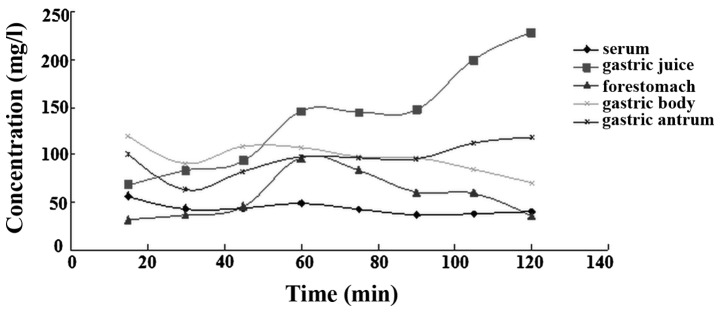
Mean concentration-time profiles of levofloxacin in the serum, gastric juice and all regions of the stomach at a 100 mg/kg dose with rabeprazole in rats.

**Figure 4:**
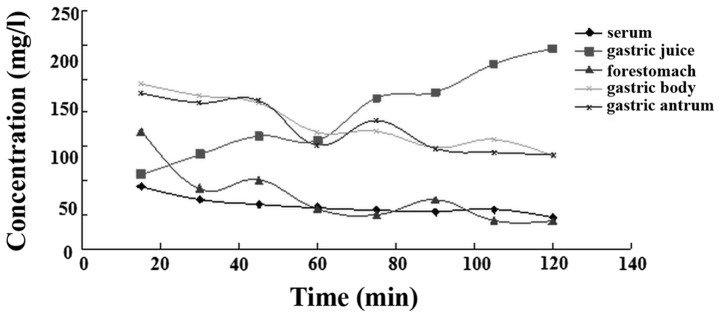
Mean concentration-time profiles of levofloxacin in the serum, gastric juice and all regions of the stomach at a 50 mg/kg dose with rabeprazole in rats.

As shown in Figs. 3 and 4, the gastric juice levofloxacin concentration was significantly higher than the serum concentration from 30 min (100 mg/kg levofloxacin + rabeprazole) and 15 min (50 mg/kg levofloxacin + rabeprazole). The levofloxacin concentration in the gastric body and gastric antrum was significantly higher than that in the serum and forestomach from 30 min (100 mg/kg levofloxacin + rabeprazole, with the exception of the 105- and 120-min time-points) and 15 min (50 mg/kg levofloxacin + rabeprazole). The levofloxacin concentration in the forestomach exhibited no significant difference from that in the serum, and no significant difference was observed in the levofloxacin concentration between the gastric body and antrum from the 15-min time-point. The stomach clearance rate of 100 mg/kg levofloxacin + rabeprazole was 1.30 l/h/kg, and the transport fraction was 2.55. The stomach clearance rate of 50 mg/kg levofloxacin + rabeprazole was 0.99 l/h/kg, and the transport fraction was 2.42.

The levofloxacin concentrations in the gastric juices, forestomach and gastric body in the 100 mg/kg levofloxacin group significantly differed from those in the 50 mg/kg levofloxacin group starting at the 90-min time-point (P<0.05), but the levofloxacin concentration in the gastric antrum exhibited no significant difference between the two groups (P>0.05). The levofloxacin concentrations in the gastric juice in the 100 mg/kg levofloxacin + rabeprazole group differed from those in the 50 mg/kg levofloxacin + rabeprazole group, except at the 60-min time-point. The levofloxacin concentrations in the forestomach, gastric body and gastric antrum differed significantly, with the exception of certain time-points (P<0.05). However, levofloxacin concentrations in the body of the stomach did not differ, with the exception of one time-point (75 min) (P>0.05).

### Effect of rabeprazole on the levofloxacin distribution

The drug concentrations in the gastric juice, forestomach, gastric body and gastric antrum in the 100 mg/kg levofloxacin + rabeprazole group at each position and at each time-point were greater than those in the 100 mg/kg levofloxacin group, although no significant difference was observed (with the exception of the concentrations in the gastric juice at 45 min, the forestomach at 15 and 120 min, the gastric body at 75 min and the gastric antrum at 75 min). The transport fraction in the stomach of the 100 mg/kg levofloxacin + rabeprazole group was slightly higher than that in the 100 mg/kg levofloxacin group, but the difference was not significant. The drug concentrations in the gastric juice, forestomach, gastric body and gastric antrum in the 50 mg/kg levofloxacin + 50 mg/kg rabeprazole group were greater than those in the 50 mg/kg levofloxacin group at each position and time-point, but the difference was not significant. The transport fraction in the stomach of the 50 mg/kg levofloxacin + rabeprazole group was slightly higher than that of the 50 mg/kg levofloxacin group, but the difference was not significant.

### Effect of stomach pH on the combination of levofloxacin with rabeprazole

Subsequent to infusing the rats with 2 mg/kg rabeprazole via a peripheral vein, their gastric pH increased rapidly at the selected time-points. The gastric pH of the majority of the rats was increased at the 30-min time-point, but other rats exhibited an increased gastric pH at the 15-min time-point, which remained high for 2 h. The pH in the rat stomach ranged from 1.3 to 3.2 (median, 2.2) when only levofloxacin was administered. Combining levofloxacin with rabeprazole caused the gastric pH to range from 1.9 to 6.8, with a median of 5.2. Analysis of the gastric pH in association with the drug concentration in the gastric juice, forestomach, gastric body, gastric antrum and glandular stomach revealed that the levofloxacin concentration increased with increasing gastric pH, but the relevant factor was not statistically significant.

## Discussion

Clinical and experimental studies on antibiotics for the classical triple-therapy regimen for *H. pylori* are available. A previous study on the transport and distribution of antibiotics in the stomach showed that amoxicillin diffusion is affected by the protein-binding rate and mucosal permeability, and that the drug concentration of gastric juice is lower than the serum concentration (18). Metronidazole easily diffuses and penetrates the gastric mucosa and rapidly reaches or even exceeds the serum concentration in the stomach due to its high hydrophobicity and the possible existence of active transport (4). Clarithromycin is more significantly affected by active transport, and is currently the most effective anti-*H. pylori* drug (7). Omeprazole affects the minimum inhibitory concentration (MIC) and chemical stability of antibiotics by increasing the pH value in the stomach, and can increase the system transport of antibiotics, such as clarithromycin, to improve the anti-*H. pylori* efficiency of antibiotics (11). These findings provide experimental data from different aspects for the classical triple-therapy regimens.

In the present study, levofloxacin at 50 mg/kg and at 100 mg/kg, alone or in combination with rabeprazole, resulted in the same overall trend in levofloxacin concentration in gastric juice and all sections of the stomach. Within the 2 h sampling time, the levofloxacin concentration in gastric juice increased with time, except during in the early stages. The levofloxacin concentration in the gastric juice was significantly higher than that in the serum between the third and fifth time-point. The levofloxacin concentration in the forestomach, gastric body and gastric antrum was high at the 15-min time-point, and subsequently decreased with time. The levofloxacin concentration in the forestomach was low and similar to the serum concentration, whereas the concentrations in the gastric body and antrum were markedly higher than those in the serum. The levofloxacin concentrations in the gastric body and in the antrum were notably higher than those in the forestomach. The levofloxacin concentration in the gastric body was slightly higher than that in the gastric antrum, but the difference was not significant, which indicates that the levofloxacin is not uniformly distributed in rat stomach tissues. The drug concentration-time curve for levofloxacin concentration in the serum and in other tissues indicated that the concentration was unusually increased at certain time-points, which is inconsistent with the theoretical concentration trend (particularly in the serum); this may have resulted from the individual differences among the rats. The transport fraction of levofloxacin in the stomach was marginally higher in the treatments in which it was combined with rabeprazole than those with levofloxacin alone, but the difference was not significant. The increase in the 50 mg/kg levofloxacin group was slightly greater than that in the 100 mg/kg levofloxacin group. Considering the trend in the drug concentration-time curve, the levofloxacin concentration in the gastric mucosa increased rapidly with the serum concentration, which indicates that the onset of levofloxacin in anti-*H. pylori* therapy is rapid and the penetrating capacity is strong. These results are consistent with those on the local concentration of levofloxacin in tissues and in other regions (19). At the selected time-points in the present study, the levofloxacin concentrations in all tissues of the gastric mucosa were higher than the MIC_50_ of *H. pylori* in China (20). The levofloxacin concentration in gastric juice at the selected time-points was higher than the MIC_50_ for *H. pylori*, which reflects the transport condition of levofloxacin, but its high concentration in the gastric juice suggests that levofloxacin affects the *H. pylori* colonisation of the gastric mucosa and gastric mucus layer through local direct action.

The changes in levofloxacin concentrations in gastric juice indicate that levofloxacin may be actively transported in the stomach, as its concentration in gastric juice was three- to five-fold that in the serum (except during the early stages), which is inconsistent with cis-concentration gradient transport (21), i.e. passive transport of the drug within the body. The levofloxacin concentration-time curve in the gastric juice was suggestive of active transport. Clarithromycin is generally considered to be actively transported to the stomach (with a transport fraction of 2.28), whereas amoxicillin undergoes little transport in the stomach (with a transport fraction of 0.15) (10). Therefore, levofloxacin may be actively transported in the stomach. Furthermore, levofloxacin is actively transported in the kidneys and the small intestines (22,23). The high transport fraction of levofloxacin in the stomach indicates a high transport capacity to the gastric juice and a strong penetrating power, which favours the eradication of *H. pylori*. Quinolones may be a promising class of anti-*H. pylori* drugs. However, further validation is required, particularly using the system in an *in vitro* study model.

The stomach has a strongly acidic environment, and antibiotics have different stabilities in acidic environments. Combining levofloxacin with rabeprazole increased the pH in the rat stomach within 15–30 min, which then remained high. Considering the changes in levofloxacin concentration in the gastric juice when levofloxacin was combined with rabeprazole in the two dose groups, the levofloxacin concentration in the gastric juice eventually exceeded that in the serum, and was consistent with the change in pH in the stomach. The levofloxacin concentration in gastric juice was slightly higher than that in the serum, but the difference was not statistically significant. Combining 50 and 100 mg/kg levofloxacin with rabeprazole slightly changed the drug concentration in each section of the stomach. In the 100 mg/kg levofloxacin + rabeprazole group, the concentration in the gastric body and antrum did not significantly differ from that in the serum at the 105–120-min time-points, which was not observed in the 50 mg/kg levofloxacin + rabeprazole group, as the concentrations in the gastric body and antrum remained higher than those in the serum. This difference may be associated with the individual differences of rats. The comparison of the levofloxacin transport fraction in the stomach in the 50 mg/kg levofloxacin, the 50 mg/kg levofloxacin + rabeprazole, the 100 mg/kg levofloxacin and the 100 mg/kg levofloxacin + rabeprazole groups revealed that rabeprazole slightly increased the transport fractions of levofloxacin, which indicates that the presence of rabeprazole or increases in the gastric pH value affect levofloxacin transport in the stomach. The effect of rabeprazole on levofloxacin concentration did not significantly differ among these groups, which may be investigated further by increasing the sample size and the number of time-points. A previous study demonstrated in a renal monolayer cell model (24) that the flow rate of levofloxacin from the basal surface to the cavity surface is greater than that from the cavity surface to the basal surface. The decrease in pH in the cavity surface lowers the flow rate of levofloxacin, and levofloxacin significantly inhibits the flow rate of tetraethylammonium. Thus, H^+^ may be involved in a reverse transport system in the levofloxacin transport. However, a previous study found that the levofloxacin transport pathway may be unassociated with the H^+^ reverse transport system (23).

The difference in levofloxacin concentration in the gastric juice between the 50 mg/kg levofloxacin and the 100 mg/kg levofloxacin groups primarily started at the 90-min time-point, which may be associated with the presence of different carriers or the involvement of a transport system in levofloxacin transport and the uneven levofloxacin distribution in the stomach. Saturation of certain carriers or the transport systems would cause differences in levofloxacin distribution. By contrast, the groups also treated with rabeprazole exhibited differences in levofloxacin concentration in the gastric juice at the all the other time-points, with the exception of the 60-min time-point, which indicates that the H^+^ reverse transport system may be involved in the levofloxacin transport in the stomach. Similarly, the differences in drug concentration between the 50 mg/kg levofloxacin and the 100 mg/kg levofloxacin groups in the forestomach and the gastric body were evident at the 90-min time-point, but no significant difference in drug concentration was observed in the gastric antrum (with the exception of a few time-points). Cotreatment with rabeprazole caused significant differences in levofloxacin concentration in the forestomach and the gastric antrum at all except a few time-points. However, other than at a few time-points, the drug concentration in the gastric body did not differ at all, which indicates that different carriers or transport systems are present in the gastric body and the antrum for levofloxacin transport in the stomach. Data on the transport and distribution of levofloxacin in the stomach are lacking, but the results of this study revealed that levofloxacin may be actively transported in the stomach. However, the putative participating transport systems still require identification. An *in vitro* study is necessary, and an appropriate transport inhibitor must be used in further studies.

In conclusion, levofloxacin may be actively transported in the stomach. The levofloxacin concentration in the gastric antrum is higher than that in the forestomach, and the local concentration increases with increasing dosage. Combining a PPI with levofloxacin has little effect on the drug concentration and distribution in the stomach within 2 h.

## Figures and Tables

**Table I:** Precision of the high-performance liquid chromatography method for the analysis of levofloxacin in the serum, gastric juice and gastric mucosa of rats.

	Between-day RSD (%)			Within-day RSD (%)		
		
Sample	Low concentration	Medium concentration	High concentration	Low concentration	Medium concentration	High concentration
Serum	2.21	2.64	0.37	4.60	3.31	0.48
Gastric juice	0.92	1.95	1.17	3.56	1.92	4.29
Gastric mucosa	1.51	1.69	3.38	1.23	3.11	1.53

RSD, relative standard deviation.

**Table II:** Stability of the high-performance liquid chromatography method for the analysis of levofloxacin in the serum, gastric juice and gastric mucosa of rats.

	Room temperature RSD (%)			−20°C RSD (%)		
		
Sample	Low concentration	Medium concentration	High concentration	Low concentration	Medium concentration	High concentration
Serum	3.27	2.95	0.52	2.56	2.42	0.71
Gastric juice	3.71	2.36	3.25	1.27	2.57	1.72
Gastric mucosa	1.92	3.01	1.59	1.33	2.61	3.19

RSD, relative standard deviation.

**Table III:** Accuracy and sensitivity of the high-performance liquid chromatography method for the analysis of levofloxacin in the serum, gastric juice and gastric mucosa of rats.

	Recovery (%)				
			
Sample	Low concentration	Medium concentration	High concentration	LOD (ng)	LOQ (ng)
Serum	81.61±1.81	102.89±2.72	100.84±0.37	5	25
Gastric juice	87.19±0.78	96.38±1.88	99.01±4.25	5	10
Gastric mucosa	88.09±1.33	97.19±1.64	99.26±3.36	10	25

LOD, limit of detection; LOQ, limit of quantification.
